# Seroprevalence of hepatitis B and C virus in HIV-1 and HIV-2 infected Gambians

**DOI:** 10.1186/1743-422X-7-230

**Published:** 2010-09-15

**Authors:** Modou Jobarteh, Marine Malfroy, Ingrid Peterson, Adam Jeng, Ramu Sarge-Njie, Abraham Alabi, Kevin Peterson, Matt Cotten, Andrew Hall, Sarah Rowland-Jones, Hilton Whittle, Richard Tedder, Assan Jaye, Maimuna Mendy

**Affiliations:** 1 Medical Research Council, Fajara, P O Box 273, Banjul, The Gambia; 2 London School of Hygiene and Tropical Medicine, Keppel Street, London, WC1E 7HT, UK; 3 National Health Protection Agency, 61 Colindale Avenue, London NW9 5EQ, UK; 4 Centre Léon Bérard 28 rue de Laennec 69373 Lyon cedex 08, France; 5 U.S Department of Defense HIV Program (Nigeria), 7 Usuma Street, Maitama, Abuja, Nigeria; 6 Weatherall Institute of Molecular Medicine, University of Oxford, John Radcliffe Hospital, Headington, Oxford OX3 9DS, UK

## Abstract

**Background:**

The prevalence of HIV/hepatitis co-infection in sub-Saharan Africa is not well documented, while both HIV and HBV are endemic in this area.

**Objective:**

The aim of this study is to determine the seroprevalence of HBV and HCV virus in HIV-infected subjects in the Gambia.

**Methods:**

Plasma samples from HIV infected patients (190 individuals with clinically defined AIDS and 382 individuals without AIDS) were tested retrospectively for the presence of HBV sero-markers and for serum HBV DNA, screened for HCV infection by testing for anti-HCV antibody and HCV RNA.

**Results:**

HBsAg prevalence in HIV-positive individuals is 12.2%. HIV/HBV co-infected individuals with CD4 count of <200 cells uL-1 have a higher HBV DNA viral load than patients with higher CD4 count (log 4.0 vs. log 2.0 DNA copies/ml, p < 0.05). Males (OR = 1.8, 95% CI: 1.0, 3.2) were more likely to be HBsAg positive than female. HCV seroprevalence was 0.9% in HIV-positive individuals.

**Conclusion:**

The prevalence of HBsAg carriage in HIV- infected Gambians is similar to that obtained in the general population. However co-infected individuals with reduced CD4 levels, indicative of AIDS had higher prevalence of HBeAg retention and elevated HBV DNA levels compared to non-AIDS patients with higher CD4 count.

## Background

It is estimated that 350 million people world -wide are chronically infected with hepatitis B virus (HBV) and over 500,000 people die annually from HBV-related causes [1,2]. HBV Carriers are at a high risk of developing cirrhotic liver disease and hepatocellular carcinoma (HCC), the most frequent cause of cancer morbidity and mortality worldwide [3]. Hepatitis C virus (HCV) produces a chronic infection in up to 80% of infected individuals. Like HBV, the virus is a major cause of severe liver fibrosis, cirrhosis and HCC [4,5]. Approximately 170 million people are infected with HCV worldwide and over three million new infections occur each year [6]. The prevalence rates in sub-Saharan Africa are highly variable, ranging from 0-40% with Cameroon having a prevalence of 13% [7] and 16% reported in pregnant women in Malawi [8,9].

Although HBV and HCV are well documented for the general Gambian population [10-13], there is limited data on HBV and HCV seroprevalence in Human Immunodeficiency Virus (HIV)-infected Gambians. HBV, HCV and HIV infections are important causes of infectious diseases worldwide. HIV affects more than 33.4 million people worldwide, of which 22.7 million live in sub-Saharan Africa and 2.7 million new HIV infections were reported in 2008 [14]. In West Africa, Acquired Immunodeficiency Syndrome (AIDS) is caused by both HIV-1 and the related but generally less pathogenic HIV-2 [15].

The prevalence of HIV-1 reported in Senegal, The Gambia and Guinea Bissau is between 0.5-5.0% [16] and that of HIV-2 is between 3.3 to 8.3% [17,18]. However, recent studies in Bissau have reported a decrease in HIV-2 from 8.3% to 4.7% in a period of 17 yrs, whilst HIV-1 is on the increase from 0.5% to 3.7% [19].

When both HBV and HIV co-infect a patient, the mortality rate from chronic hepatitis B is increased above that of either infection alone with a faster rate of progression to liver cirrhosis and hepatocellular carcinoma (HCC) [20-22]. Co-infected individuals have a reduction of HBV surface antigen (HBsAg) seroconversion, higher levels of HBV DNA and often show reactivation of HBV replication despite previous HBsAg seroconversion [23].

In this era of rolling out Highly Active Antiretroviral Therapy (HAART) it is important to document HIV-HBV co-infection in regions with high chronic hepatitis B endemicity and HIV infection rates. In the U.S. liver disease, due to chronic HBV and/or HCV infection, has become one of the leading causes of mortality among people with HIV infection, despite the low prevalence in the general population. Moreover, some ARVs, including lamivudine (3TC) commonly used in first line ART, possess anti-HBV activity. When these drugs are used as monotherapy for HBV treatment, this will create the potential for inducing HBV viral drug resistance mutations and selection of viral populations that may escape current HBV vaccines.

The aim of this study is to determine the prevalence of HBV and HCV in HIV infected subjects and to compare the level of HBV DNA, a marker of HBV replication in AIDS vs. non-AIDS patients.

## Results

### Demographic data and HIV status of subjects in the study

The demography data is presented in Tables 1 and 2. The age ranges of the subjects were 7 months -71 years (median = 35 yrs) for AIDS patients and 17 - 93 yrs (median = 31 years) for non-AIDS subjects. The proportions of females infected with HIV were 61% in the AIDS and 80% in the non-AIDS cohort. Overall, HIV-1, HIV-2 and HIV-Dual infections accounted for 52%, 43% and 5% of HIV infections. However, HIV-1 infection made up 75% of the HIV infections in the AIDS cohort, compared to only 41% in the non-AIDS. Median CD4 count at baseline was significantly lower in the AIDS patients at pre-treatment time point compared to the non-AIDS (p-values in each HIV-strata <0.001, analysis not shown). The CD4 values did not vary significantly across HIV-type in either the AIDS patients (pre-treatment time point) or non-AIDS groups (AIDS cohort, p-value = 0.55; non-AIDS cohort, p-value = 0.36; analysis not shown). In the AIDS cohort, median HIV viral loads before the start of treatment were not different between HIV type (5.1 log_10 _copies mL-1 for HIV-1 and 4.8 l_og10 _copies mL-1 for HIV-2 (p-value = 0.28).

**Table 1:** Baseline characteristics of HIV-infected patients at MRC Genito-Urinary Clinic, the Gambia

	AIDS patients (pre-treatment time point)	Non-AIDS	Total
**Gender^1^**	**N = 190**	**N = 382**	**N = 572**
Male	74 (39.3)	77 (20.1)	151 (26.3)
Female	116 (60.7)	305 (79.8)	421 (73.6)

**Age^1^**			
0-9 years	27 (14.2)	0 (0.0)	27 (4.7)
10-24 years	10 (5.2)	84 (21.9)	94 (16.4)
25-34 years	30 (15.7)	183 (47.9)	213 (37.2)
35-44 years	72 (37.8)	62 (16.2)	134 (22.5)
45-93 years^	51 (14.2)	53 (13.8)	104 (3.4)

**HIV Status^1^**			
HIV-1	142 (74.8)	157 (40.9)	299 (55.2)
HIV-2	29 (15.2)	215 (56.4)	244 (42.6)

**Dual Infection**	19 (10.0)	10 (2.6)	29 (5.0)

^1 ^Number and percent are reported for gender, age, HIV status.

^^ ^Maximum age in AIDS was 71 years; maximum age in non-AIDS was 93 years

**Table 2:** Baseline HIV viral load and CD4 counts of HIV-infected patients at MRC Genito-Urinary Clinic, the Gambia

	*AIDS*	*Non-AIDS*	*Total*	
	*(pre-treatment)*	*(baseline)*		
**HIV-1 Infection**	**N = 142**	**N = 157**	**N = 299**	
^1^Viral Load (c mL-1)	1.27 × 10^5 ^(6.9 × 10^5^)	--	--	
^1 ^CD4 Count (cells μL-1)	*160 (220.0)	*690.0 (510.0)	390.0 (600.0)	
CD4 Count < 200 cells μL-1^2^	78 (56.9)	5 (3.2)	83 (28.3)	
^1^CD4 Percent	7 (9.0)	32.0 (8.0)	15.5 (24.0)	

**HIV-2 Infection**	**N = 29**	**N = 215**		
^1^Viral Load (c mL-1)	5.88 × 10^4 ^(3.5 × 10^5^)	--		
^1^CD4 Count (cells μL-1)	**140 (210.0)	**649.0 (450.0)	600.0 (510.0)	
^2^CD4 Count < 200 cells μL-1	17 (58.6)	9 (4.2)	26 (10.7)	
^1^CD4 Percent	10 (14.0)	34.0 (10.0)	33.0 (11.0)	

**HIV-1 and HIV-2**	**N = 19**	**N = 10**		
^1^HIV-1 Viral Load (c mL-1)	1.60 × 10^5 ^(3.4 x10^5^)	--		
^1^HIV-2 Viral Load (c mL-1)	100 (5.8 × 10^3^)	--		
^1^CD4 Count	140.0 (130.0)	**720.0 (282.0)	180.0 (488.0)	
^2^CD4 Count < 200 cells μL-1	14.0 (77.8)	0 (0)	14 (51.9)	
^1^CD4 Percent	8.0 (6.0)	33 (6.0)	9.0 (8.0)	

**ALT for all HIV +**				
^1^ALT Level	***20.0 (14.0)	***14.0 (10.5)	16.0 (12.0)	
^1^Abnormal (ALT > 46)	15.0 (7.6)	7.0 (2.9)	22 (5.1)	

^1 ^Median values are reported for HIV viral load, CD4 count, CD4 percent, ALT level. HIV viral load was not measured in non-AIDS. Viral load units are copies mL-1 (c mL-1). ^2^Number and percent are reported for CD4 count <200 and abnormal ALT. *10 HIV-1 (9 AIDS, 1 non-AIDS) and 2 Dual (1 AIDS, 1 non-AIDS) were not tested for CD4 count; **53 HIV-2 (1 AIDS, 52 non-AIDS) and 5 Duals in the non-AIDS group were not tested for CD4 percent. ***In total, 160 patients (14 AIDS and 146 non-AIDS) were not tested for ALT.

### HBV infection in HIV infected Gambians

Overall 78.1% (447 out of 572) of HIV positive individuals tested either positive for HBsAg or anti-HBc. Seventy samples tested positive for HBsAg, giving an overall prevalence of chronic HBV of 12.2% (95% CI [0.09 - 0.15]) (Table 3). HBsAg prevalence did not vary significantly between AIDS and non-AIDS groups (15.7% vs. 11%) (p-value = 0.29, analysis not shown). Additionally, univariate analysis showed no significant differences in HBsAg prevalence by gender, age group, HIV type or baseline CD4 cell count. However a logistic model which regressed HBsAg on age, sex, HIV-type and immune status revealed that HIV infected males were significantly more likely to be HBsAg positive (OR = 1.8, 95% Confidence Interval [CI]: 1.0, 3.2) than women, as were younger people (10-24 yrs) compared to adults (OR [per year] = 1.9. 95% CI: 0.9, 1.0) (Logistic analysis is not shown in the table).

**Table 3:** HBV Seromarker prevalence by demographic and HIV status

	AIDS patients Pre-treatment time-point			Non-AIDS Baseline		
**Positive HBV Sero Markers**	**HBsAg**	**HBeAg^1 ^**	**HBV-infected ^2 ^**	**HBsAg N (%)**	**HBeAg^1 ^**	**HBV-infected 2 N (%)**
	**N (%)**	**N (%)**	**N (%)**	**N (%)**	**N (%)**	**N (%)**
**Sex:**						
Male	13 (17.6)	6 (46.2)	63 (86.3)	11 (14.3)	0 (0.0)	62 (87.3)
Female	16 (13.8)	4 (25.0)	95 (77.8)	30 (9.8)	8 (26.6)	227 (83.2)
P-value difference	0.48	0.23	0.14	0.26	0.17	0.39

**Age:**						
10-24 years	1 (10.0)	1 (100.0)	10 (58.8)*	14 (16.9)	4 (28.6)	65 (83.3)
25-34 years	11 (19.3)	4 (36.4)	47 (92.2) *	17 (9.3)	2 (12.5)	135 (82.8)
35-44 years	13 (18.0)	4 (30.8)	59 (88.1) *	4 (6.4)	1 (25.0)	48 (82.7)
45-93 years	4 (7.8)	1 (25.0)	39 (84.8) *	6 (11.3)	1 (16.7)	42 (93.3)
P-value difference	0.31	0.54	< 0.0001	0.17	0.71	0.36

**HIV status:**						
HIV-1	25 (17.6)	9 (36.0)	117 (78.5)	16 (10.1)	3 (18.8)	109 (76.8)*
HIV-2	3 (10.3)	0 (0.0)	24 (85.7)	24 (11.1)	4 (17.4)	174 (89.2)*
Dual Infection	1 (5.3)	1 (100.0)	17 (99.4)	1 (11.1)	1 (100.0)	7 (87.5)*
P-value difference	0.27	0.17	0.21	0.96	0.29	0.008

**CD4:**						
< 200 cells μL-1^3^	14 (13.4)	5 (31.2)	87 (84.5)*			
> 200 cells μL-1	11 (18.9)	3 (27.2)	51 (71.8)*			
P-value difference	0.35	0.82	0.04			

**Total**	**29 (15.3)**	**10 (34.5)**	**158 (81.0)**	**41 (10.7)**	**8 (20.0)**	**290 (84.1)**

^1 ^HBeAg status assessed in individuals who were HBsAg positive, ^2 ^Subjects who were HBsAg positive or HBcAb positive were considered to by HBV-infected. 164 (90%) of 183 HBsAg negative AIDS patients and 302 (89%) of 345 HBsAg negative non-AIDS individuals were tested for anti-HBc, 42 non-AIDS patients and 31 AIDS patients were HBsAg positive; 122 (74.5%) and 247 (98%) respectively had a positive anti-HBc result. ^3 ^189 AIDS patients had CD4 measurements, 109 had values <200 cell μL-1. * Chi-Square, Fisher's exact, Rank Sum or Kruskal-Wallis p-value <0.05; all but 14 of the non-AIDS patients had CD4 counts > 200 cells μL-1 25

Overall, 26.1% (95% CI [16.2. 36.5]) of chronic carriers were HBeAg positive, this did not differ by clinical status; i.e. AIDS vs. non-AIDS (p-value = 0.17) but HBeAg positivity was associated with HIV type as 14.8% (4/27), 30% (12/40) and 100% (2/2) HIV-2, HIV-1 and Duals respectively tested positive for HBeAg (p-value = 0.03).

The overall prevalence of anti-HBc antibody in the 502 HBsAg negative HIV-infected individuals was 79.1 (95% CI [79.0, 86.1], the marker showed an increase with age; however this trend was not statistically significant. In multivariable logistic analysis, only male gender and HIV type were significantly associated with HBcAb positivity. In the model, HBcAb prevalence was significantly higher in men (OR = 2.2, 95% CI [1.2, 4.3]) (The logistic analysis is not shown).

Overall, twenty-five of seventy HBsAg positive individuals had detectable HBV DNA, 62% (18/29) of AIDS and 17% (7/41) of the non-AIDS carriers (Table 4) with higher prevalence observed in HIV-1 and HIV-Dually infected patients compared to HIV-2 (43.2% and 100% vs. 20.8% respectively) (p-value 0.03). In paired t-tests these differences were significant between HIV-1 and HIV-2 and between HIV-2 and HIV-Dually infected patients, but not between HIV-1 and HIV-Duals (analysis not shown). A higher proportion of men in the AIDS group had detectable HBV DNA at pre treatment time point than their women counterparts (83.3% vs. 40.0%) (p-value 0.05), but this trend was not observed in the non-AIDS group at the baseline time point.

**Table 4:** HBV DNA Status in HBsAg positive subjects by demographic and HIV status

	AIDS (pretreatment time point) (N = 29)		non-AIDS (N = 41) *		Total (N = 66)	
**HBV DNA**	**Detection**	**Geometric**	**Detection**	**Geometric**	**Detection**	**Geometric**
	**N (%)**	**Mean (c mL-1)**	**N (%)**	**Mean (c mL-1)**	**N (%)**	**Mean(c mL-1)**
**Sex:**						
Female	7 (43.7)*	2.3 × 10^2^	7 (26.9)	6.2 × 10^3^	14 (29.2)	1.2 × 10^4^
P-value difference	0.05	0.04	0.07	0.07	0.20	0.64

**Age**						
0-9 years	1 (100.0)	27.2	--	--	1 (100.0)	2.7 × 10^1^
10-24 years	1 (100.0)	6.7 × 10^2^	4 (28.5)	1.1 × 10^4^	5 (29.4)	6.3 × 10^3^
25-34 years	5 (50.0)	2.9 × 10^2^	1 (7.1)	6.6 × 10^3^	6 (21.3)	4.9 × 10^2^
35-44 years	9 (69.2)	1.9 × 10^2^	0 (0.0)	--	9 (52.9)	1.9 × 10^2^
45-93 years	2 (50.0)	2.2 × 10^3^	2 (33.3)	1.9 × 10^3^	4 (40.0)	2.0 × 10^3^
P-value difference	0.83	0.81	0.31	0.30	0.12	0.53

**HIV Status**						
HIV-1	14 (58.3)	2.2 × 10^2^	3 (21.4)	1.1 × 10^4^	17 (40.4)*	4.3 × 10^2^
HIV-2	2 (66.2)	1.3 × 10^3^	3 (14.2)	2.9 × 10^3^	5 (18.5)*	2.1 × 10^3^
Dual Infection	2 (100.0)	2.4 × 10^2^	1 (50.0)	1.2 × 10^4^	3 (75.0)*	8.7 × 10^2^
P-value difference	0.77	0.48	0.37	0.34	0.003	0.09

**CD4 Count**						
< 200 cells μL-1**	11 (73.3)	2.6 × 10^2^	--	--	--	--
> 200 cells μL-1	5 (34.4)	1.8 × 10^2^	--	--	--	--
P-value difference	0.22	0.19	--	--	--	--

**Total**	**18 (62.0)**	**2.6 × 10^2^**	**7 (16.6)**	**6.2 × 10^3^**	**25 (34.2)**	**6.5 × 10^2^**

*18 (62%) out of 29 AIDS patients and 7 (16.6%) out of 42 non-AIDS HBsAg individuals tested positive for HBV DNA.

** Number and percent are reported for CD4 count <200.

### CD4 levels in HBV-HIV co-infected individuals

CD4 counts were obtained for 184 out of 190 AIDS patients at prior to ART including 29 HBsAg positive of which 25 had CD4 counts (Table 3). The individuals were divided into two groups based on their CD4 count (cut off < 200 cells μL-1). The prevalence of HBsAg positivity was not associated with CD4 levels, with equal proportion of HBsAg positive subjects reported in either the low level or high level CD4 group (11/29 vs. 14/29) (Table 3). Co-infected patients with low CD4 count (< 200 cells μL-1) had a higher HBV DNA viral load than patients with high CD4 count of (> 200 cells μL-1) (2.5 × 10^4 ^vs. 2.8 × 10^2 ^DNA copies mL-1) (p < 0.05).

### HCV infection in HIV infected Gambians

Two independent HCV antibody assays result in 19.4% (37/190) in AIDS and 6.7% (26/382) in non-AIDS individuals testing positive for HCV (Table 5). However, confirmatory test using RIBA HCV 3.0 SIA detected only 2 (1.0%) positive samples from the AIDS and 5 (1.3%) from the non-AIDS group, 56 samples were not confirmed by RIBA of which 5 showed indeterminate results, The age of these 7 individuals ranged from 29 to 68 years and they were all negative for HBsAg (Table 6).

**Table 5:** Anti-HCV seroprevalence and HCV RNA in AIDS and non-Aids individuals

	AIDS (N = 190)	Non-AIDS (N = 382)
*ELISA positive	37 (19.4%)	26 (6.8%)
**RIBA positive	2/37 (5.4%)	5/26 (19.2%)
***RT-PCR positive	1/37 (2.7%)	3/26 (11.5%)

* The ELISA positive individuals in the AIDS group include 24/142 (16.9%) HIV-1, 6/29 (20.1%) HIV- 2 and 7/19) (36.8%) HIV-Duals compared to 8/157 (5.0%) HIV-1, 18/215 (8.3%) HIV- 2 and none HIV-Duals in the non-AIDS group.

** HCV antibody test was confirmed in 1 AIDS and 3 non-AIDS patients in the HIV-2 group and in 1 AIDS and 2 non-AIDS in the HIV-1 group

***50% (1/1) and 60% (3/5) RIBA positive samples had detectable HCV-RNA.

5 samples (4 AIDS and 1 non-AIDS) had indeterminate result by ELISA but were negative for RT-PCR

**Table 6:** Characteristics of 7 individuals co-infected with HCV

Patient ID	Age (yrs)	Study group	Gender	HIV type	CD4 count (Cells/μL)	HCV- RNA
1	46	Non-AIDS	Male	HIV-2	500	Positive
2	55	Non-AIDS	Male	HIV-2	580	Positive
3	37	Non-AIDS	Male	HIV-1	550	Positive
4	68	AIDS	Male	HIV-2	90	Positive
5	56	Non-AIDS	Male	HIV-2	350	Negative
6	29	Non-AIDS	Female	HIV-1	700	Negative
7	36	AIDS	Female	HIV-1	85	Negative

All samples were anti-HCV positive by RIBA

HCV RNA detection by RT-PCR was performed in order to determine the prevalence of chronic HCV infection. Using primers specific to the 5'UTR and NS5b regions we amplified 251 bp and 379 bp fragments respectively. HCV RNA was detected in 4 (1- AIDS and 3- non-AIDS) out of 7 RIBA HCV 3.0 SIA positive samples and in none of the 56 RIBA negative samples. Genome sequence data from the 4 HCV RNA positive samples were compared with sequences from the GenBank. Phylogenetic analysis on the Gambian HCV sequences in comparison with the GenBank sequences showed similarity with HCV genotype 2 sequences AF037254, AF037239 and AF037253(data not shown).

## Discussions

HIV-HBV Dual infection is not uncommon where both diseases are endemic. We assessed the level and impact of this co-infection among both AIDS and non-AIDS patients. Comparing these infections provided an insight into the role of co-infection in disease progression in chronic HBV carriers [23].

The HBsAg prevalence detected in HIV infected individuals was 12.2% with 78.1% positivity for either HBsAg or anti-HBc, which is comparable to the overall levels obtained in children [10,11] and in controls from a liver cancer case control study [12]. 62% of the children were infected with HBV with between 17-36% HBsAg positivity and highest rates of HBsAg carriage was reported in the younger children. The controls in the Kirk et al., study consisted of mainly adults with no liver related disease. Since The Gambia has low levels of HIV infection, with reported rates of 1-3% in the general population [24], the similarity of HBV prevalence reported in the previous studies and in the HIV- positive population in our study suggests that people infected with HIV do not have greater exposure or susceptibility to HBV than the general population.

Unlike the situation in the U.S. and Europe, HBV in sub Saharan Africa is commonly transmitted during childhood between siblings, typically long before infection with HIV [25], Burkina Faso [26] and Cote d'Ivoire Coast [25,27-29]. Over 30% of co-infected HBsAg carriers >25 yrs old were positive for hepatitis B e antigen (HBeAg), this is greater than the rate reported in similar age group in a non-HIV population of which the adult HBV carriers were found to be in the inactive carrier phase [30]. This is the third phase of chronic hepatitis B that is traditionally identified by the absence of HBeAg and HBV DNA for potentially indefinite duration. Thus similar to reports from other African studies, HBe antibody seroconversion occurred less frequently in Gambian HIV-infected individuals suggesting that HIV infection either delayed transition to the inactive carrier phase [31-34] or facilitate re-emergence of HBV replication. This has serious implications as studies have shown that patients who test positive for HBeAg and/or raised HBV DNA are those who are at highest risk of developing advanced liver disease [35,36].

The degree of immunodeficiency represents an important factor in the progression of hepatitis among individuals co-infected with HBV and/or HCV [37]. There is the risk of reactivation of chronic hepatitis B in HBV, sometimes referred to as reverse seroconversion [31], and occult hepatitis B. Occult hepatitis, defined by undetectable serum HBsAg combined with measurable serum HBV DNA, may be associated with progression to cirrhosis and HCC [38] in co-infected patients. It is anticipated that the natural history of HBV will change in sub-Saharan Africa as more countries introduce infant vaccination; this is likely to influence the rate of HBV-HIV co-infection in the future. In The Gambia HBV vaccination is done in infancy, the first dose given between the ages of 1 and 4 weeks, with a coverage rate of >80% [39]. However universal vaccination was introduced only 19 years ago so subjects in the current study over 19 years old would not have had the opportunity to be vaccinated.

The HCV seroprevalence of 0.9% RIBA positive was lower than the frequency reported in other countries in Africa of 1.6-6.0% [7]. Although HCV had been found to be of low prevalence in the Gambia, a surprisingly high frequency of 19.0% was once reported in HCC patients from a HCC case control study [12]. In our study 7 out 572 HIV positive individuals were co-infected with HCV and this was observed exclusively in the older individuals and none of them were HBV carriers.

The age distribution of HCV infection was previously reported in a Gambian study of HIV negative individuals and a cohort effect was proposed as a possible reason for this finding [12]. Our study confirmed the presence of genotype 2, similar to the findings in Guinea Conakry and Guinea Bissau [40,41]. However like the Ruggieri et al., study, we showed heterogeneity in subtype clustering. Similar low rate of HCV-HBV co-infection was also reported in a previous study in Gambian patients referred for HIV screening [13]. The high rate of false positive with the ELISA test could be due to amino acid sequence variability and purity of the HCV antigen used in the assays. The sensitivity and specificity of the HCV ELISA have been shown to be influenced by high immunoglobulin G (IgG) concentration of human blood [42]. The lack of amplification with the 3 anti-HCV positive samples may be ascribed to a low viral RNA content or to virus degradation.

The over representation of HIV-1 in the AIDS group (over 5-fold higher than HIV-2) compared to the non-AIDS group is consistent with previous reports of a longer median time to AIDS in HIV-2 with a comparatively smaller proportion of HIV-2 infected patients developing AIDS

We observed a striking gender difference between the two HIV groups registering a female to male ratio of over 2.5. Since this was a clinic based study, in the absence of incidence data, it is unclear whether the gender distribution of HIV infection or HBV/HCV co-infection reflects the general population. However, these results are similar to a report from a national population-based HIV prevalence surveys conducted in 19 countries in sub-Saharan Africa, which showed a predominance of females in the HIV infected groups, with the lowest female: male ratio reported at 2.2[43,44]. Despite the over representation of women in the two HIV positive groups, a higher proportion of HIV positive men had detectable HBV DNA compared to their female counterparts, suggesting a higher level of viral activity which could lead to higher rate of liver disease in males. This findings complements results from previous studies that showed higher proportion of men (male: female sex ratio around 2.4) with advanced liver diseases compared to women [12]. Despite the difference in gender distribution of hepatocellular carcinoma especially in men in high-risk geographical areas, there is little documented evidence for sex-linked differences in HBV replication [45].

In conclusion, we showed that the prevalence of HBV chronic infection in HIV positive subjects in the Gambia was similar to that found in the general population. Co-infection with HIV however can lead to higher frequency of HBeAg positivity and higher levels of HBV DNA indicating higher levels of HBV replication.

Studies on the impact of HIV infection in the natural history of chronic HBV and the effect of chronic hepatitis B on immune recovery are necessary. The question as to whether there is a lower or delayed increase of CD4 lymphocyte count in HIV/HBV co-infected patients on ART is currently being investigated by our group. The current study recommends HBsAg screening for HIV patients before the start of ART.

This work was supported by Medical research Council (The Gambia) and with a small grant from Professor Richard Tedder.

## Materials and methods

### Subjects

This retrospective study was conducted in two groups of HIV infected patients recruited from the Genito-Urinary Medicine (GUM) clinic from 1988 to 2008 and ARV treatment started in 2004. During the period of 2005 to date, the vast majority of GUM clinic patients are from the general population presenting to the Medical Research Council (MRC) directly. They are often self referrals with symptoms of sexually transmitted illness and between 10-25% of them get tested for HIV as a result of medical illness. Prior to 2004, when there was no active HIV screening nationwide the proportion of self referrals to MRC were still as high as 85%-90%.

The first study group (AIDS), consists of 190 HIV infected individuals with clinically-defined AIDS according to WHO criteria of clinical stage IV and or a CD4 < 200 cells μL-1. The second group (non-AIDS) consists of 382 HIV-infected individuals without clinical stage IV and had baseline CD4 counts > 200 cells μL-1 with the majority having > 350 cells μL-1. Pre treatment and baseline samples from the AIDS and non AIDS group respectively were tested for HBV serology and HBsAg positive samples had HBV DNA measurement. Samples were tested for HCV seromarkers and HCV genotype determined by sequencing of the 5'UTR and NS5b regions. The AIDS patients had CD4 count, HIV viral load and ALT results whilst non-AIDS patients had CD4 count and ALT results.

Ethical approval was granted by the joint Gambia Government/MRC Ethics Committee. All subjects and/or legal guardians provided written, informed consent.

### HIV Serology, CD4 cell counts and ALT measurement

HIV-1 and HIV-2 infections were screened for HIV antibodies using combined enzyme-linked immuoabsorbent assay (ELISA) (Abbott Murex HIV 1.2.0 test kit, Murex Diagnostics Ltd, Dartford, UK) and confirmed by a 2 type-specific ELISA and synthetic peptide-based strip method, Pepti-Lav 1-2 (Sanofi Diagnostics Pasteur, Marne la Coquette, France) and dilutional assays [46]. CD4 cell count measurement was performed by flow cytometry (Becton-Dickinson, Belgium) and plasma HIV-1 and HIV-2 viral load measurement was done using an in-house viral load assay [47] ALT was measured using Roche Cobas Mira Chemistry Analyzer.

### Hepatitis B virus serology

HBsAg test was by immunochromatography (Abbot Determine ™), HBsAg positive samples were further tested for Hepatitis B e antigen (HBeAg) and antibodies (anti-HBe) by ELISA (DiaSorin, Sallugia, Italy). All HBsAg negative samples were subjected to Hepatitis B core antibody (anti-HBc) test using ELISA (DiaSorin).

### HBV DNA quantification

DNA was extracted from HBsAg positive samples using QIAamp DNA Mini Kit (Qiagen, UK) and quantified using real time PCR with HBV specific primers as previously described and utilizing primers HBV TAQ 1 (GTG TCT GCG GCG TTT TATCA) and HBV TAQ-2 (GAC AAA CGG GCA ACA TAC CTT) for the amplification [48].

### Hepatitis C Virus serology, RNA detection and sequencing

Samples were screened for HCV antibodies using an anti-HCV ELISA kit, (Abbott Murex, version 4.0). All positive samples were rescreened using the same ELISA. Samples that were repeatedly positive were confirmed using a recombinant immunoblot assay (Chiron RIBA HCV 3.0 SIA, Chiron Corporation).

All of the HCV-antibody positive samples, including those that tested negative for RT-PCR were further tested for the presence of HCV RNA by reverse transcription PCR (RT-PCR) and nested PCR using primers specific to the 5'untranslated region (5'-UTR) and non-structural (NS5b) regions of HCV. Prior to RT-PCR, RNA was extracted from plasma using QIAmp viral RNA reagents (Qiagen). The RT-PCR mixture containing 300 ng RNA, 0.4 mM dNTP (each), 0.2 μM of primers NF5 (sense GTGAGGAACTACTGTCTTCACGCAG) and NR5 (antisense TGCTCATGGTGCACGGTCTACGAGA) was subjected to one cycle of RT at 50°C followed by 30 cycles of PCR to amplify the 5'UTR region followed by a second round PCR amplification [49]. Similarly, the NS5b region was amplified by performing two round of PCR using two sets of primers ^4^EF101F-TTCTCGTATGATACCCGCTGTTTTGA and HCVNS5RnB-TACCTGGTCATAGCCTCCGTGAAGGCTC [41]. Gel purified PCR products were sequenced using primers specific for the 5'UTR (KF2 - TTCACGCAGAAAGCGTCTAG and 211-CACTCTCGAGCACCCTATCAGGCAGT) and NS5b (HCVNS5F2-TATGATACCCGCTGCTTTGACTCG; HCVNS5R2c-CTGGTCATAGCCTCCGTGAAGGCTCTCAGG and HCVNS5R2d-CTGGTCATAGCCTCCGTGAAGGCTCGTAGG

### Statistical Analysis

HBV seroprevalence was determined in the two HIV positive groups. To identify factors associated with the presence and severity of HBV infection, univariate analysis was conducted to assess HBV seroprevalence for HBsAg, HBeAg and HBcAb and the geometric mean and median of HBV DNA copies across demographic and HIV-related variables. Analyses were stratified by cohort membership in the AIDS and non-AIDS patients. Statistically significant differences in HBV seromarker prevalence was assessed by Chi-Square or Fisher's exact tests. The difference in the geometric mean and median HBV DNA copies was assessed by the Kruskal Wallis test. Multivariable logistic regression models were then developed to examine the impact of demographic and HIV type on prevalence of HBsAg, HBeAg and HBcAb. Factors which were statistically significant in univariate analysis or which were of theoretic interest were included in the full model. A logistic model that regressed HBsAg on age, sex and HIV-type was run. All analyses were carried out in SAS 9.1 (SAS Institute, Cary, North Carolina).

## Competing interests

The authors declare that they have no competing interests.

## Authors' contributions

Conceived and designed the study: MM, MLJ, MMF. Analyzed the data: IP, ML, MM. Contributed to the assembling of the longitudinal HIV cohort: HW, AJ, RSN, AA, SRJ, KP. Contributed to the drafting the manuscript: MLJ, MMF, IP, AJB, RSN, AA, KP, MC, AH, SRJ, HW, RT, AJ. MM wrote the paper: MLJ, MM. All authors read and approved the final version of the manuscript.
